# Postural adaptation to microgravity underlies fine motor impairment in astronauts’ speech

**DOI:** 10.1038/s41598-023-34854-w

**Published:** 2023-05-22

**Authors:** Arian Shamei, Márton Sóskuthy, Ian Stavness, Bryan Gick

**Affiliations:** 1 University of British Columbia, Vancouver, Canada; 2 University of Saskatchewan, Saskatoon, Canada; 3 Haskins Laboratories, New Haven, CT USA

**Keywords:** Motor control, Sensorimotor processing

## Abstract

Understanding the role of anti-gravity behaviour in fine motor control is crucial to achieving a unified theory of motor control. We compare speech from astronauts before and immediately after microgravity exposure to evaluate the role of anti-gravity posture during fine motor skills. Here we show a generalized lowering of vowel space after space travel, which suggests a generalized postural shift of the articulators. Biomechanical modelling of gravitational effects on the vocal tract supports this analysis—the jaw and tongue are pulled down in 1g, but movement trajectories of the tongue are otherwise unaffected. These results demonstrate the role of anti-gravity posture in fine motor behaviour and provide a basis for the unification of motor control models across domains.

## Introduction

Gravity plays a central role in models of body posture^1,2^, and by extension, in general models of motor control^3^. It has long been understood that posture largely consists of tonic muscle activations that serve anti-gravity functions^2^. Alterations to gross motor skills such as gait and limb control observed in astronauts following gravitational change are taken to represent changes to anti-gravity behaviour^4–6^. While anti-gravity posture is understood to play a critical role in gross motor skills, its role in fine motor skills is not often discussed despite evidence of gravity-related adaptation during fine motor control^7–11^, highlighting a significant point of divergence across different domains of motor control. Clarifying the role of anti-gravity posture in fine motor skills is a missing key in achieving a unified theory of movement control.

Speech recordings from astronauts provide a valuable source of data to assess the role of posture in fine motor skills by allowing comparisons of vocal tract posture and articulatory precision immediately before and after adaptation to microgravity. The acoustic signal of speech is determined by movements of the tongue, jaw, lips and larynx; for example, it is well understood that specific vocal tract resonances known as vowel formants correspond to specific positions of the tongue and jaw^12,13^. Here we show a generalized lowering of speech articulators after space travel, reflecting a generalized postural shift of the articulators. Biomechanical modelling of the vocal tract in 1g and 0g conditions further supports this analysis - the jaw and tongue are pulled down, but movement trajectories are otherwise unaffected. These results demonstrate the role of anti-gravity posture in fine motor behaviour and provide a basis for the unification of motor control models across domains.

### Fine motor skills and anti-gravity behaviour

While it is widely understood that anti-gravity behaviour such as posture is integral for movement^3^, discussions of fine motor skills often omit gravity because its effect on movements is small and difficult to measure. For example, speech is one of the most complex human fine motor skills, yet current models of speech motor control are purely kinematic controllers, and do not account for external forces such as gravity or anti-gravity postural control^14^. Previous work investigating the effect of gravitational load on tongue and jaw movements found that that jaw occlusion was altered based on the rotation of the head relative to gravity, and that vowel formants were also measurably different. This was taken to indicate that neural control signals of the jaw and tongue do not completely offset external forces such as gravity^15–17^. However, this work did not account for longer term adaptation effects in which anti-gravity behaviour can manifest.

Speech data presents an ideal context for assessing the role of anti-gravity behaviour in fine motor skills for two main reasons: (1) there exists abundant speech data from spaceflight missions across different gravitational conditions; (2) speech data allows for precise comparisons of fine motor skills from movements that are both natural and highly complex. The present study compares speech from astronauts before and immediately after prolonged exposure to microgravity. In doing so, we aim to observe whether and how vocal tract musculature adapts to microgravity environments. Using publicly available audio files, we compare tongue height via vowel formants from four astronauts immediately before and after NASA Space Shuttle (STS) missions. We employ linear mixed effects modelling and biomechanical modelling of vowel productions to determine the effects of gravity on tongue and jaw kinematics before and after adaptation to microgravity.

After returning from the Expedition 35 space mission, astronaut Chris Hadfield reported:

“I could feel the weight of my lips and tongue and had to change how I was talking... I didn’t realize I had learned to talk with a weightless tongue”^18^.

Hadfield’s comments suggest that adaptation to changing gravity conditions can have a substantial effect on speech. Astronauts who spend extended durations in space adapt their movements and posture for microgravity, and must later re-adapt motor control for Earth gravity^4,6,19^. This reflects both human’s sophisticated internal model of gravity^5^ and the adaptability of that internal model to changing gravity conditions^6^. Considering mechanical alteration to the speech production system is known to affect speech perception as well^20^, it stands to reason that the ability to both send and receive spoken information may be compromised during transitions in gravity, which are often critical to the mission, and provides further support for observations that gravitational transitions may broadly impair fine motor skills in astronauts^11^.

Although previous work in fine motor control has often skirted the anti-gravity question, a number of studies have suggested that a postural substrate of some kind is needed for speech. For example, Perkell (1969) described a ”pre-speech posture” upon which articulatory movements are superimposed^21^; Gick et al. (2004) provided instrumental data showing that speakers of different languages use distinct postural configurations of the vocal tract, even during non-speech periods between utterances^22^. There is evidence that speakers actively raise and hold the tongue against the molars in a distinct posture known as lingual bracing, serving as a mechanical and proprioceptive anchor^23^. The present study uses speech as a context for assessing whether postural substrates of this kind constitute evidence of an anti-gravity substrate underlying fine motor skills.

As a preliminary test of Hadfield’s observations, the present authors conducted a preflight and postflight comparison of tongue height using data from the STS-134 mission^24^. A distinct lowering of the vowel space was observed postflight, however, the small dataset lacked appropriate statistical power, and did not include methodology to identify the source of tongue lowering. For example, it is unclear whether the baseline position of the jaw and tongue were affected, or just their respective movement trajectories. The former would indicate anti-gravity postural behaviour, the latter would not. While this work provided empirical support for Hadfield’s observations, it provided no insight into the role of anti-gravity behaviour during fine motor control. The present analysis substantially expands upon this work with the inclusion of additional astronauts for appropriate statistical power, and additional experiments to determine the source of tongue lowering between conditions.

In order to test the general hypothesis that antigravity postural substrates underlie fine motor skills, the present paper tests the specific hypothesis that speakers in 1g (Earth) conditions maintain an anti-gravity postural substrate in the vocal tract. Under this hypothesis, adaptation to microgravity (space) conditions will result in motor plans which do not account for the downward force imposed by 1g. We predict a generalized lowering of the speech articulators upon re-entry to 1g which would result in a measurable lowering of the acoustic vowel space, represented as an increase of the first formant (F1) across vowels.

If the predicted effect on tongue height is observed, it is important to distinguish whether this effect stems from alterations to postural configurations of the tongue and jaw or from changes to individual movements. We employ two methods to assist in interpreting the source of observed adaptations: First, we include vowel height as a fixed effect within our model to examine the consistency of any change throughout the vowel space: Vowels are categorized phonetically as high or low based on differences in their tongue height and jaw occlusion. A gravity-induced shift in the baseline position of articulators should affect high and low vowels similarly regardless of their movement direction. If, in contrast, only movement trajectories of the tongue and jaw are affected by gravitational change, then we would expect vowels that are produced higher in the mouth to show greater lowering after gravitational change by virtue of having greater upward movements^25,26^.

Second, we include 3D biomechanical modelling of the tongue and jaw during articulation and rest in microgravity and 1g conditions to test whether the observed transitions reflect a global shift in the positions of articulators versus a localized change to their movement trajectories. We conduct two pairs of simulations (four total) incrementally decoupling the predicted effects of gravity on the position of articulators (jaw vs. tongue) from its effect on their vertical movement trajectories. In each pair of simulations, the first simulation evaluates the effect of gravity on the baseline position of a specific articulator (jaw or tongue) at rest. This is done by comparing the distance between specified points on the speech articulators (e.g., tracking the upper and lower jaw, tongue body and dorsum). The second simulation in each pair repeats these measurements following vertical movement (i.e., jaw abduction, tongue raising). Comparing the results of the simulations in each pair allows us to decouple the effects of gravity on the baseline position of jaw and on its movements trajectories. If gravity enhances downward movement trajectories of the jaw, or upward movement trajectories of the tongue, the difference in movement distance between gravitational conditions should be larger than the difference observed cross-conditionally at rest.

## Materials and methods

### Formant analysis

The authors declare that the data used within this analysis was obtained from publicly available sources, and all research was completed in accordance with human research ethics guidelines and regulations. The National Aeronautics and Space Administration (NASA) provides audio and video for a selection of missions through the public NASA images archive, which contains audio and video files of Astronaut interviews for selected missions^27,28^ Audio files from each mission were selected because they feature substantial speech data (between 70 and 120 s of speech) of astronauts immediately before and after their respective missions, allowing direct comparisons of vowel space before and after adaptations to microgravity. We evaluated vowel formants from four astronauts across four STS missions: (i) STS-129 (ii) STS-133, (iii) STS-134, and (iv) STS-135. Our dataset was limited to the captain of each respective flight, as immediate post-flight interviews are only provided for the captain of each flight. In all cases, subjects were male. This dataset represents the complete set of Space Shuttle program ISS missions for which there are available postflight interviews to make such a comparison. Each mission ranged from 10 to 14 days in length^29^ aboard the International Space Station. Exposure to microgravity aboard the ISS for this duration is known to produce gravity-related sensorimotor adaptation effects throughout the body^6,30^.

For each astronaut, data from two conditions (pre-flight interview and post-flight tarmac interview) were manually transcribed and then assessed using semi-automated alignment and formant extraction via FAVE-Extract^31^ and the Montreal Forced Aligner^32^ using the DARLA web interface^33^ and the Vowels R package^34^. Stopwords (e.g. function words such as *and*, *the*, *but*) were omitted from the analysis (for a complete list of all omitted stopwords see http://darla.dartmouth.edu/stopwords) along with unstressed vowels, to ensure that the measurements represent full vowels only. Tokens where the formant bandwidth exceeded 300Hz were excluded to ensure accurate identification of the target vowel.

As our hypothesis is focused on the effects of gravity and tongue height, we have limited our report in the present paper to F1. We used non-normalized F1 values returned by DARLA for full vowels. Formant measurements were taken at three points for each vowel: at 35%, 50%, and 65%. We report results based on the average of these three measurements, allowing us to smooth over temporary irregularities in the formant measurements for monophthongs, and to include at least some information about the full trajectory of diphthongs. Formant values reported by DARLA were examined manually for impossible values, of which none were observed. Formant values were then converted to their corresponding mel scale values using the emuR package in R^35^, allowing us to analyse formant values on a scale that is more in line with human auditory perception^36^. Each vowel was assigned a binary height value (high/low). For height, near-open and open vowels were classed as low, near-closed and closed vowels were classed as high. Tokens containing the vowels /ɔ, ʊ, aʊ,ɔI/ were omitted as these vowels lacked a sufficient number of tokens for comparison. Table 1 provides the binary height classification for each vowel, along with the number of tokens for each speaker per condition.

A variable-slope linear mixed-effect model evaluating F1 was fit to the data in R^37^ using the lme4 package^38^ and the optimx optimizer^39^. The model was designed to assess the effect of condition on F1 while accommodating our hypothesis regarding high and low vowels. Our model included random intercepts by speaker and vowel; random slopes over condition, height and their interaction by speaker; and random slopes over condition by vowel. The corresponding lme4 formula in R is as follows:
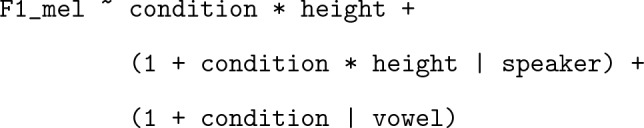

**Table 1:** Vowel metadata and token quantity for each speaker and condition.

Token		CF		CH		MK		SL		Total	
V	Lo	Pre	Post	Pre	Post	Pre	Post	Pre	Post	Pre	Post
a	+	6	6	7	5	2	1	6	10	21	22
æ	+	9	10	6	11	9	8	6	22	30	51
ʌ	+	13	15	7	12	5	5	10	10	35	42
a	+	8	6	8	10	5	3	8	14	29	33
ɛ	+	14	13	17	5	8	16	9	23	48	57
e	−	8	14	10	19	8	14	17	12	43	59
I	−	21	13	17	13	13	14	18	22	69	62
i	−	10	12	15	8	8	10	4	15	37	45
o	−	11	14	6	6	7	2	5	9	29	31
u	−	15	7	6	6	4	2	12	4	37	19

### Biomechanical simulations

We evaluated the effects of gravity on the tongue and jaw using the biomechanical simulation platform Artisynth^40^. We used a three-dimensional computational model of the vocal tract incorporating coupled tongue-jaw-hyoid dynamics and finite-element musculature; a detailed description of the model can be found in^41^. This model has the advantage of incorporating gravity as a controllable parameter, allowing us to compare biomechanics of specific muscle activations of the tongue and jaw in simulated 1g and 0g environments. We note that while 0g is not present within our solar system, comparing the effects of simulated 0g and 1g allows us to remove the effects of gravity on the vocal tract in their entirety, which provides a more salient comparison of the effects of gravity on vocal tract structures.

Incorporation of biomechanical simulations provides insight into the source of change, namely, whether any shift in the vowel space is consistent with a general postural shift. A grid with 0.5cm cell width was overlaid on the model; The vertical distance in pixels was measured between the start and end point for each articulation, and converted into centimetres in relation to the overlaid grid, allowing measurements of tongue and jaw position with controlled activations in microgravity and 1g environments. We present four simulations in total.

#### Simulation 1 & 2

Simulation 1 compares passive vertical displacement of the jaw (and by extension, the tongue) from rest in 0g and 1g, which allows us to model the extent to which gravity passively lowers the jaw from rest. We measured the vertical distance between specified target points on a) the upper and lower jaw and b) the dorsum of the tongue and center of the palate. The vertical distance between target points was compared between conditions. Simulation 2 evaluates the effect of gravity on downward movement trajectories of the jaw following controlled activation of the bilateral jaw opening muscles. Using the same target points from simulation 1, vertical distance was measured following 5% activation of the bilateral jaw opening musculature and the resulting displacement from rest was compared between 0g and 1g. Further comparing the results of simulations 1 and 2 allow us to decouple the effects of gravity on passive lowering of the jaw from gravitational enhancement of downward jaw movement.

#### Simulation 3 & 4

In simulations 3 and 4, the jaw is locked in place, and the effects of gravity on passive lowering and active raising of the tongue is compared without interference from jaw displacement. The vertical distance between the dorsum of the tongue and the center of the palate was measured, with the resulting distance from rest in 0g and 1g compared. Simulation 3 compared passive displacement (lowering) of the tongue, with no muscle activation provided to the model. Simulation 4 compared active raising of the tongue with 15% activation of the superior longitudinal muscle. Comparing the results of simulation 3 and 4 allows us to decouple the effects of gravity on general tongue position from gravitational impedance of upward tongue movement.

## Results

### Formant analysis

**Figure 1:**
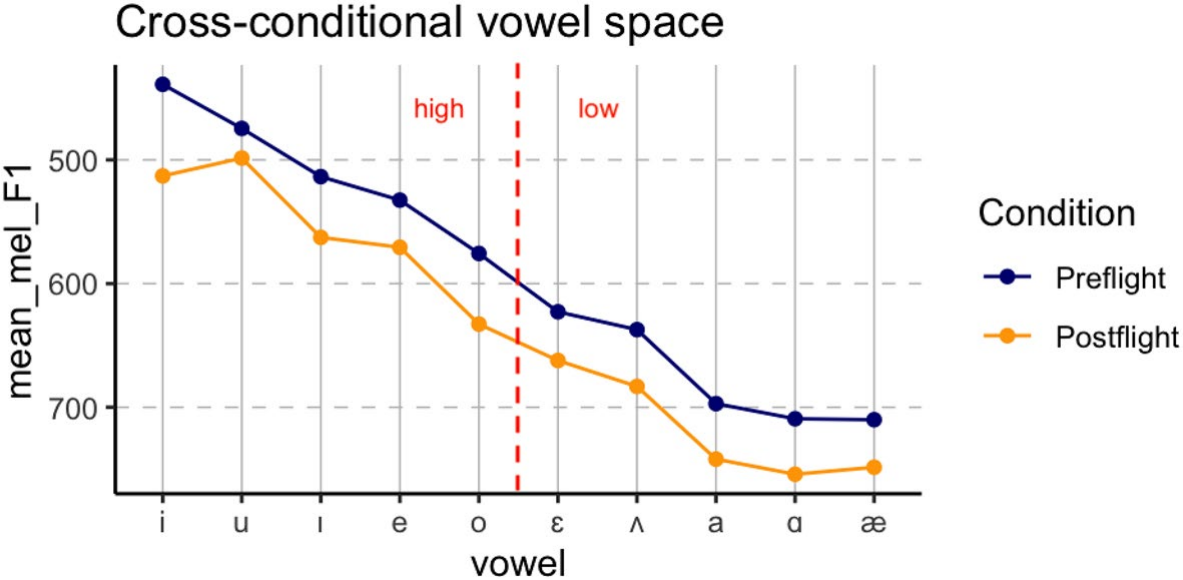
Cross-conditional vowel plot showing preflight (blue) and postflight (orange) F1 measurements averaged across the four astronauts.

Figure 1 illustrates the mean cross-conditional change in F1 for each vowel, as plotted directly from the mel-scaled raw data. Mel-scaled F1 is provided on the Y axis, with the specific vowel indicated on the X axis in ascending order of preflight F1. For each vowel, mean preflight values are indicated in blue, and postflight values in orange. A red-dotted line indicates the division between high and low vowels. Qualitatively, a consistent lowering can be observed for postflight vowels regardless of high or low classification.

#### Linear mixed effects modelling

**Figure 2:**
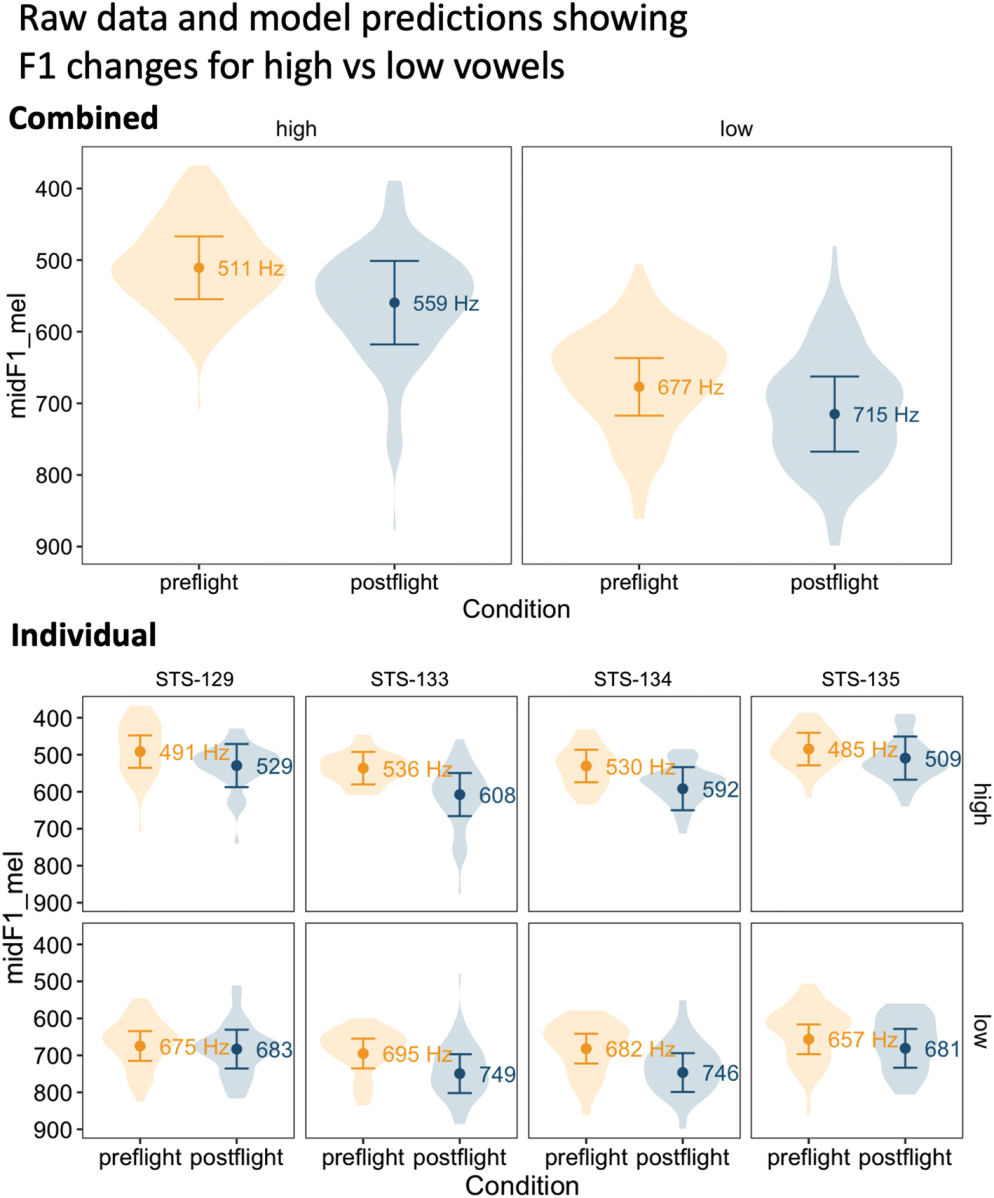
The combined effects of height and condition on F1 in the dataset. The dots, whiskers and numbers show predictions from the mixed model reported in the main text, while the violins show the distribution of the raw data.

Results for the model fitted to the mel F1 values are provided in Fig. 2, which superimposes model predictions and 95% confidence intervals on the raw data. Results are presented for all speakers in the top plot, and for individual speakers below. The figure shows the effect of condition (preflight vs. postflight) separately for high and low vowels. An LRT-based model comparison between our full model and one that omits condition entirely (including both the main effect and the interaction term) shows that condition has a significant overall effect on F1 ($\chi ^2$ = 7.33, *df* = 2, *p* = 0.025).ss An additional model comparison evaluating the interaction of height and condition found no significant interaction ($chi^2$ = 0.76, *df* = 1, *p* = 0.38). In other words, we found strong evidence that vowels are articulated lower postflight, but no evidence to suggest that high and low vowels differ in the effect of condition.

### Biomechanical simulations

**Figure 3:**
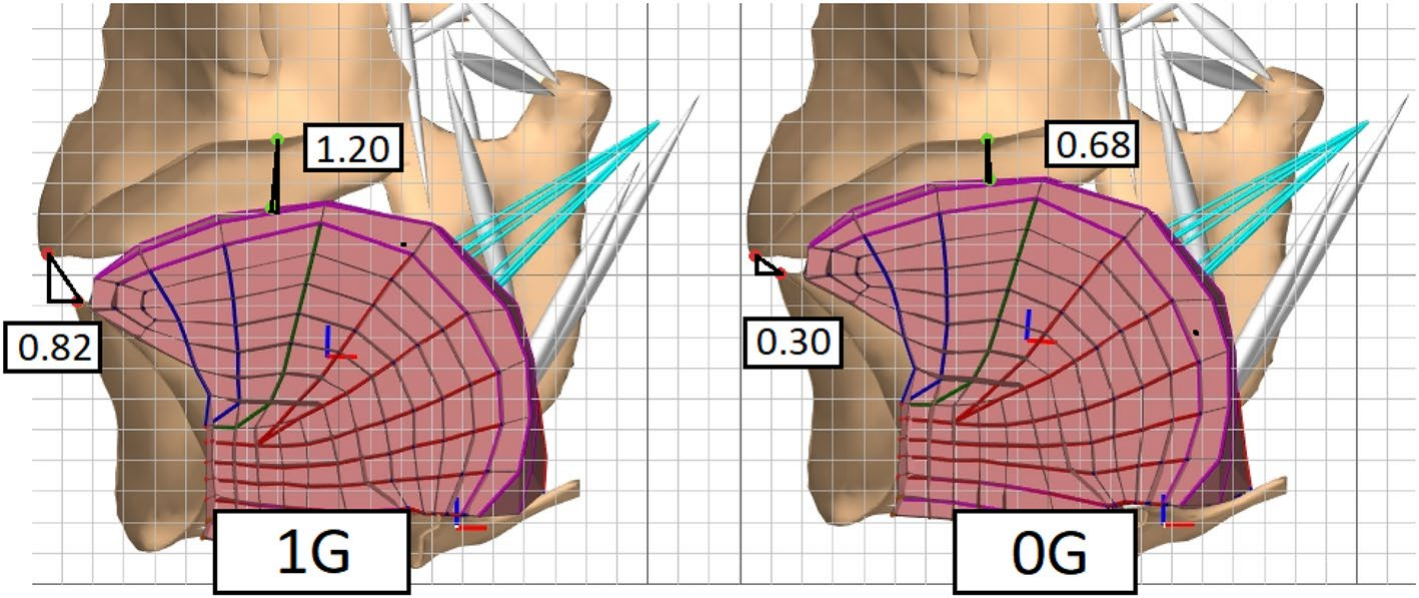
Simulation 1: Evaluating the effects of gravity on a simulated vocal tract by comparing jaw occlusion at rest in a 1g environment versus a “0g” environment. Note that in the 1g simulation, the jaw and tongue are substantially lower.

The results for all simulations are summarized in table 2. Results for each simulation are summarized below. Figure 3 provides a midsagittal view of simulation 1 with measurements of vertical distance (in cm) for each target point. The body of the tongue is shown in pink, and the hard structures (Mandible, maxilla, palate) are shown in tan. Blue bands represent muscles of the tongue, and white bands represent muscles of the jaw and hyoid.

**Table 2:** Measurements of vertical distance organized per row by simulation. Each column denotes the locus of measurement. In each cell, vertical movement distance (cm) is provided for 1G on the left, and 0G on the right.

	Jaw lowering	Tongue-palate
**Sim 1**	0.82 / 0.30	1.20 / 0.68
**Sim 2**	1.02 / 0.53	1.49 / 1.01
**Sim 3**	–	1.10 / 0.88
**Sim 4**	–	0.82 / 0.62

#### Simulation 1 & 2

In simulation 1, both the tongue and jaw were approximately 0.5 cm lower in the 1g condition relative to 0g (1g: 0.82 cm, 0g: 0.30 cm). In simulation 2, following activation of jaw abductors, the tongue and jaw were also .5 cm lower in 1g relative to 0g (1g: 1.02 cm, 0g: 0.53 cm). However, in simulation 2 any discrepancy in distance travelled disappears once we factor in the 0.5 cm difference in rest position observed in simulation 1. In other words, we found no evidence that downward jaw movement trajectories were enhanced by gravity beyond the initial passive lowering of the articulators observed in simulation 1.

#### Simulation 3 & 4

In simulation 3, the tongue at rest was approximately 0.2 cm lower in 1g relative to to 0g (1g: 1.10 cm, 0g: 0.88 cm). Following active raising of the tongue in simulation 4, the tongue was 0.2 cm lower in 1g relative to 0g (1g: 0.82 cm, 0g: 0.62 cm). Note that the .2 cm discrepancy in simulation 2 is accounted for by the .2 cm passive lowering observed in simulation 1. Therefore, we found no evidence that upward movement trajectories of the tongue are impeded by gravity beyond the initial passive lowering of the tongue in simulation 3.

## Discussion

The results of our acoustic analysis and biomechanical simulations support the hypothesis that anti-gravity postural substrates are actively employed in the vocal tract under 1g (Earth) conditions. Mixed effects models fitted to the acoustic data show that vowels were articulated significantly lower postflight, with no significant difference in outcome between high and low vowels. Overall, the acoustic findings corroborate the expectation that speakers adapt to microgravity conditions, resulting in impairments to speech upon returning to 1g. While the current study does not evaluate readaptation to 1g, and thus cannot infer the longevity of the observed changes, recent work on manual fine motor skills in astronauts following long-term adaptation to microgravity and subsequent gravitational transition to 1g, demonstrates that some level of impairment can still be observed after 30 days^11^. However, note that the astronauts in Holden’s work remained on the ISS for 6 months, thus achieving “deep adaptation” to microgravity^11^. In the present study, astronauts spent between 10 to 14 days aboard the ISS, much lower than the 90 day threshold necessary to achieve deep adaptation. Therefore, while no direct observations can be made in the present study regarding long-term effects, Holden’s work suggests that fine motor skills may be subject to long-term changes with sufficient exposure to microgravity. The present findings also suggest that gravitational transitions have the potential to affect speech communication in astronauts, similar concerns were recently raised by^11^ regarding manual fine motor skills and the control of computer systems.

Our biomechanical simulations provide further evidence that the shifted vowel space we report is due to a passive, global lowering of the tongue and jaw in 1g environments. The first pair of simulations demonstrated that gravity does not enhance downward movement trajectories during jaw abduction, but that the baseline position of the jaw is passively lowered by gravity. Likewise, the second pair of simulations found no evidence that gravity impedes upward movement of the tongue, but did demonstrate that the tongue is passively lowered at rest in 1g.

Together, the results of our vowel formant analysis and biomechanical modelling demonstrate that anti-gravity tonic muscle activations are maintained as an anti-gravity measure under 1g conditions, serving as a postural substrate in the vocal tract during speech. Adaptation to microgravity results in jaw and tongue postures optimized for microgravity, but results in altered vocal tract posture and fine motor skills upon return to Earth gravity. We note that previous works have reported alterations to fine motor skills in astronauts following exposure to gravitational change, however, to our knowledge, impaired fine motor skills following gravitational change had not been linked to underlying changes in tonic anti-gravity muscle activation, or in simpler terms, posture. We note microgravity exposure has been linked to biomechanical changes in fluid dynamics in the head and vocal tract structures – resulting in a “headward” shift of fluids^42^. Further research is necessary to distinguish the effects of fluid shifts and tonic muscle activation on speech following gravitational transitions.

Typically, posture and the role of gravity in human motor control are concepts relegated to discussions of gross motor skills such as standing, gait, balance, and limb control, where movements are large and the effects of gravity are salient. The role of anti-gravity behaviour in fine motor skills is easily ignored as the effect on movements trajectories is small, as our biomechanical simulations reflect. However, we observe that optimal execution of a complex fine motor behaviour such as speech begins with the necessary anti-gravity behaviour for the task. This has implications for models of fine motor skill, that do not incorporate gravity or anti-gravity behaviour in planning. The observation that anti-gravity behaviour is fundamental to the execution of fine motor skill presents an advancement in achieving a unified theory of motor control.

## Data Availability

Our acoustic data (formant values) and all necessary code for the statistical analyses reported in this paper are available as part of the following OSF repository: https://osf.io/mgyfd/.
