# Publisher Correction: Postural adaptation to microgravity underlies fine motor impairment in astronauts’ speech

**DOI:** 10.1038/s41598-023-36792-z

**Published:** 2023-06-14

**Authors:** Arian Shamei, Márton Sóskuthy, Ian Stavness, Bryan Gick

**Affiliations:** 1 University of British Columbia, Vancouver, Canada; 2 University of Saskatchewan, Saskatoon, Canada; 3 Haskins Laboratories, New Haven, CT USA

Correction to:*Scientific Reports* https://doi.org/10.1038/s41598-023-34854-w, published online 22 May 2023

The original version of this Article contained an error.

In the Discussion section,

“In the present study, astronauts spent between 10 and 4 days aboard the ISS, much lower than the 90 day threshold necessary to achieve deep adaptation.”

now reads:

“In the present study, astronauts spent between 10 to 14 days aboard the ISS, much lower than the 90 day threshold necessary to achieve deep adaptation.”

The original Article has been corrected.

